# Proximal Fibula Resection for Tumors—Case Series and Technical Note

**DOI:** 10.3390/jcm13237138

**Published:** 2024-11-25

**Authors:** Reinhard Hofer, Marisa Valentini, Maria Anna Smolle, Andreas Leithner, Marko Bergovec

**Affiliations:** 1 Department of Orthopaedics and Traumatology, Medical University of Graz, Auenbruggerplatz 5, 8036 Graz, Austria; reinhard.hofer@uniklinikum.kages.at (R.H.); andreas.leithner@medunigraz.at (A.L.); 2 Department of Orthopaedics and Traumatology Krems, University Hospital Krems, Mitterweg 10, 3500 Krems an der Donau, Austria

**Keywords:** proximal fibula resection, bone tumor, stability, strength, posterolateral corner, level of evidence: IV, case series

## Abstract

**Background**: The resection of tumors of the proximal fibula includes the removal of the lateral collateral ligament (LCL) and biceps femoris tendon (BFT) attachment. The aim was to describe and evaluate a surgical reconstruction technique in terms of functional outcome and knee joint stability. **Methods**: We analyzed the outcome of six patients, treated by a proximal fibula resection. The reconstruction of the attachments of the LCL and BFT was performed with two suture anchors, fixed onto the tibia at the level of the proximal tibiofibular joint (PTFJ). The postoperative knee flexion strength as well as the lateral knee joint stability were compared to the contralateral side using a digital scale and stress X-ray. Patient-reported outcome measures and postoperative complications were documented. **Results**: No lateral instability and no significant loss of knee flexion strength could be observed (*p* = 0.075). One persistent postoperative peroneus paresis was reported. High functional outcome was achieved with a mean MSTS score of 92.2%. **Conclusions**: The resection of the proximal fibula with the reinsertion of the LCL and the BFT using bone anchors in the PTFJ seems to provide a good functional outcome, with a low level of associated comorbidities.

## 1. Introduction

**Figure 1:**
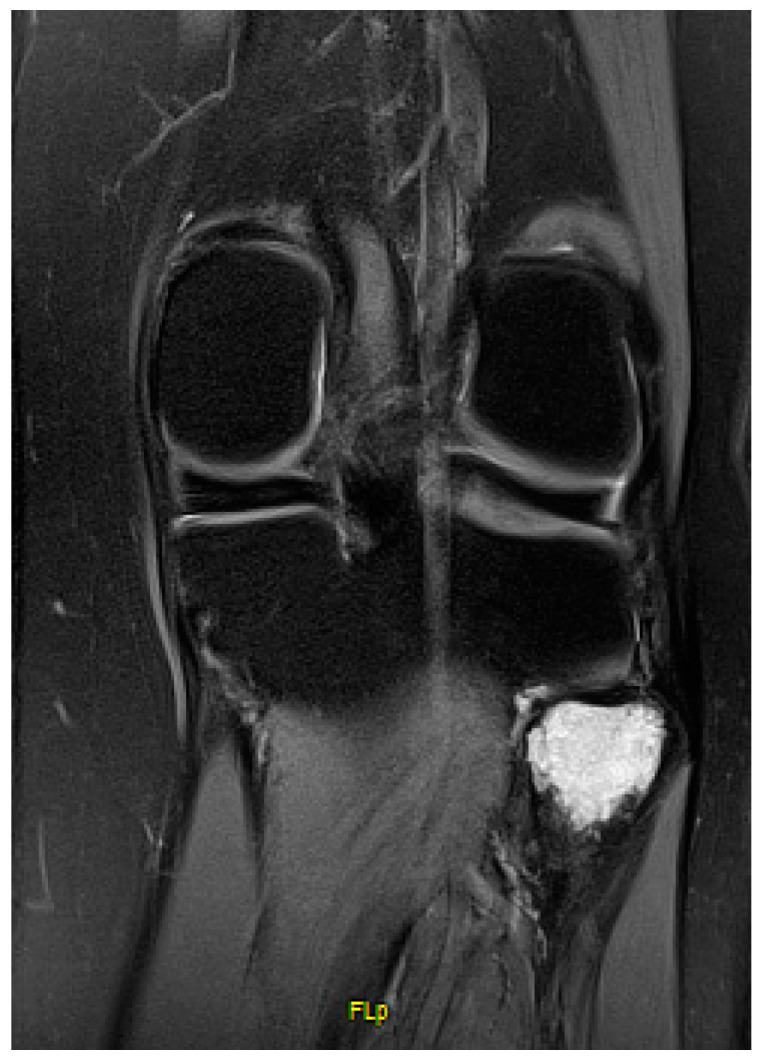
Preoperative T2 sequence of an ACT located in the proximal fibula.

The fibula is a rare anatomical location for primary bone tumors, with approximately 2.5% of tumors being located there [1]. Giant cell tumors (GCT), aneurysmal bone cysts, atypical cartilaginous tumors (ACT), and osteosarcomas are the most common types of tumors to occur, whereas the proximal fibula is the most affected site [1,2] (Figure 1). The fibula is generally considered a non-essential bone, allowing for a wider range of surgical approaches compared to other sites. For non-aggressive tumors, intralesional excision and curettage are commonly used. Adjuvant techniques, such as heat-generating PMMA cement, phenol, or other cytotoxic agents, are often used to further reduce the risk of recurrence. Aggressive or malignant tumors often necessitate an en bloc resection of the proximal fibula in the proximal tibiofibular joint (PTFJ) [3,4,5]. Malawer et al. described two types of fibula resection: Malawer type I resection is suitable for benign aggressive and low-grade malignant tumors, typically involving the removal of 2–3 cm of the fibula diaphysis as well as the attachment sites of the lateral collateral ligament (LCL) and the biceps femoris tendon (BFT). The common peroneal nerve as well as the anterior tibial artery are preserved. A Malawer type II resection is a broader extra compartmental procedure, sacrificing the common peroneal nerve, and is specifically reserved for high-grade sarcomas that invade adjacent muscle groups [5]. Surgical challenges are given not only by the close anatomical relation of the common peroneal nerve but also by the superficial part of the posterolateral corner: the LCL as a major stabilizer against varus stress and posterolateral rotation, and the biceps femoris tendon (BFT) is a major flexor of the lower leg, generating between 30% and 85% of strength during knee flexion. Injuries to the biceps femoris muscle have been associated with decreased flexion force and the rotational instability of the knee [6,7]. The surgical reconstruction after proximal fibula resection is not standardized and when not reconstructed properly, injuries of the posterolateral corner may result in instability, functional deficits, and pre-arthrotic changes [8,9,10]. We present a case series using a single surgical technique applying suture anchors to reattach the LCL and BFT at the level of the former PTFJ in patients undergoing a Malawer type I resection. The study’s aims were to evaluate postoperative lateral knee stability, to report on clinical outcomes, and to demonstrate whether reattaching the BFT results in decreased knee flexion strength compared to the contralateral side.

## 2. Methods

A total of six patients, who underwent a proximal fibula resection Malawar type 1 at our department between January 2019 and December 2022, were included for further examination. The operations were performed by a single surgeon at our department. This study was approved by the local ethics committee (34-111 ex 21/22 1550-2021).

### 2.1. Surgical Technique

**Figure 2:**
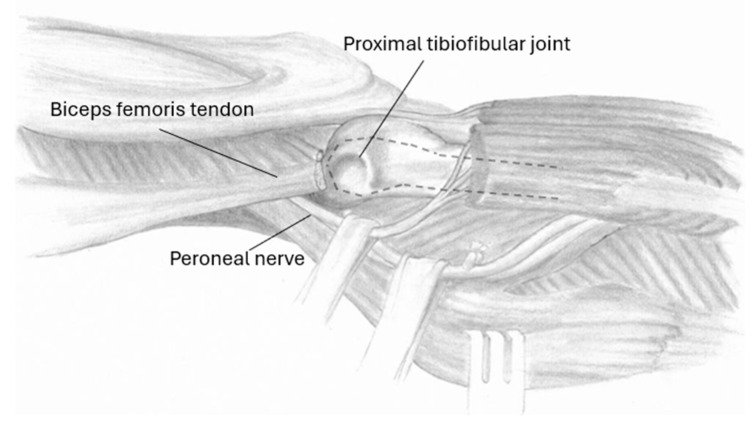
Operative situs with the PTFJ exposed for further preparation.

The patient was put in the lateral decubitus position. A single incision was made at the proximal lateral aspect of the lower leg. Dissection was carried along the BFT as well as the fibula diaphysis in between the anterior and lateral muscle compartment. The peroneal nerve was dissected and protected. The BFT as well as the LCL were dissected and detached from the fibular head and prepared for further reconstruction. Under fluoroscopy, the desired level of resection was marked, and the fibula was osteotomized and extracted in a proximal direction in order to protect the Nervus peroneus communis. The specimen was sent for histology analysis. The tibial cartilage of the PTFJ was removed in a similar fashion to a footprint preparation in a supraspinatus-tendon refixation to enhance ingrowth [11] (Figure 2). The LCL and the BFT were reinserted in 20°–30° knee flexion using two suture anchors implanted on the level of the former PTFJ (*G II Anchor*^TM^*, Depuy Synthes, Johnson & Johnson, New Brunswick, NJ, USA*). A blood-clotting sponge (*Tabotamp Fibrillar*^TM^*, Johnson & Johnson*) as well as a drainage device were inserted into the defect. Postoperative measures included full-weight bearing with crutches and a knee orthosis in extension with progressive range of motion (ROM) for six weeks. Two patients with postoperative peroneus paresis were initially treated with non-weightbearing, peroneal splinting, and knee extension for six weeks prior to progressive ROM and mobilization. All patients where included in our oncological follow-up protocol undergoing regular MRI scans.

### 2.2. Clinical Evaluations

Patients underwent clinical examination, including the testing of anterior and posterior cruciate ligaments as well as varus and valgus stress testing. The integrity of the tested structures was also controlled and documented via carried out MRIs. Additionally, patients were asked to fill out a questionnaire on functional outcome via the Musculoskeletal Tumor Society Score (MSTS), which is displayed in percent in Table 1.

### 2.3. Stability Testing

**Figure 3:**
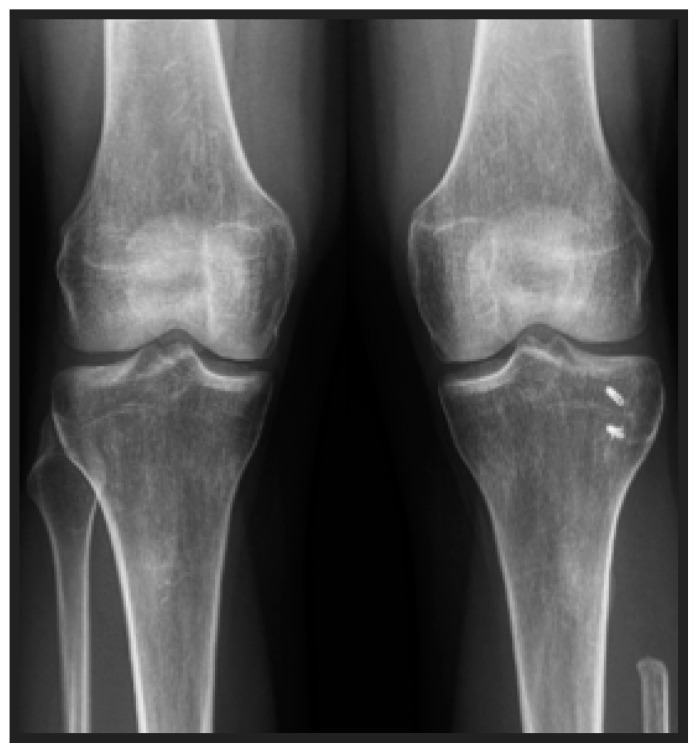
The operated knee and the contralateral side, in a neutral position.

**Figure 4:**
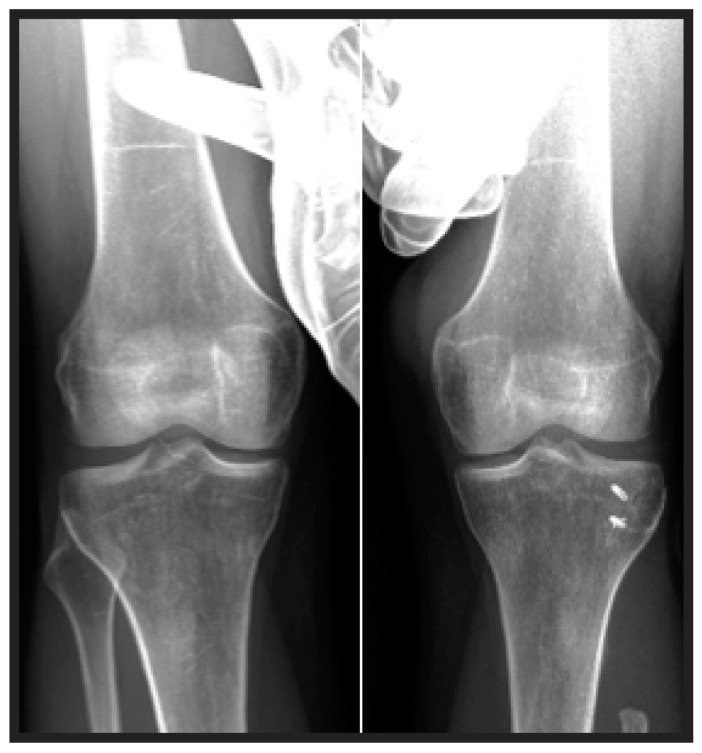
Varus stress testing of the operated and contralateral knee.

Lateral knee stability was assessed by carrying out stress radiographs, applying varus force, and measuring the degree of lateral joint space opening. The degree of instability was described via the modified Hughston Classification [12]: grade 1, lateral joint opening of 0–5 mm; grade 2, 6–10 mm; and grade 3, ≥11 mm lateral joint opening. The grade of instability was determined by comparing the results of the stress radiograph with an additional stress radiograph of the contralateral non-operated knee (Figure 3 and Figure 4).

### 2.4. Flexion-Strength Testing

**Figure 5:**
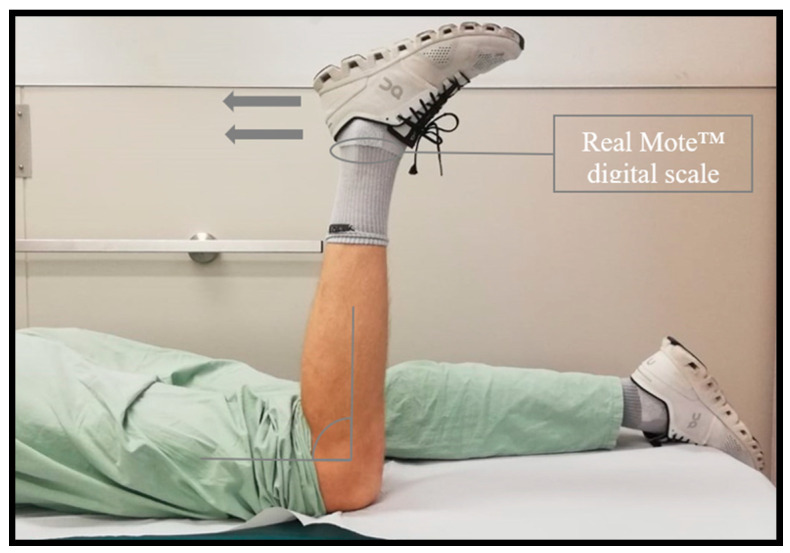
Knee flexion strength measured in prone position via a digital scale.

The strength of the knee flexion was measured with the patient being in a prone position and the knee flexed at 90°. A digital scaling device (*RealMote*^TM^*, digital scale, China Xtabarya*) was applied at the patient’s ankle, measuring the force of knee flexion in kilograms (Figure 5). The same process was repeated for the contralateral non-operated knee. The measurement for both knees was repeated 3 times, whereas the highest values were compared and displayed as a ratio in percent (Table 1). Statistical evaluation between the flexion strength of the operated and non-operated knee was performed using the paired *t*-test (*SPSS Statistics, IBM Corp., Version 23, Statistics for Windows, Armonk, NY, USA: IBM Corp.*).

## 3. Results

A total of six patients, four females and two males, were included in the present study. Atypical cartilaginous tumors (ACTs) were resected in four cases, and giant cell tumors of bone (GCTs) in two patients. The mean age at surgery was 39.2 years (range, 22–59 years), with a mean follow-up of 19.2 months postoperatively (range, 14–29 months). Preoperatively, all patients presented with pain and without signs of peroneal compression. The mean MSTS at latest follow-up was 27.6 out of 30 (92.2%). The mean knee flexion strength of the operated limb was 90.6% (81.8–103.4%) when compared to the contralateral healthy side. Applying the paired *t*-test to knee flexion strength, no significant loss of strength could be detected in between the operated and the healthy leg (*p* = 0.075). No knee instability was detected as the carried out varus stress tests showed a joint space opening within grade 1, which is identical to the joint space opening of the contralateral not-operated-on knee. Two patients (33%) reported peroneus paresis postoperatively; one of them recovered completely within 11 months, and the second patient still required peroneal splinting at last follow-up (14 months). No further postoperative complications where detected, and patients continued to be free of recurrent disease at the last oncological follow-up, with regular MRI scans being carried out.

## 4. Discussion

The resection of tumors involving the proximal fibula can potentially cause knee instability and decreased strength in knee flexion. Due to the complexity of anatomical structures and the biomechanics of the posterolateral corner, it has been referred to as “The Dark Side of the Knee” and represents a limiting factor in choosing surgical reconstruction techniques [3,13]. Our results suggest that anchoring the LCL and BFT to the former PTFJ in a surgical resection technique that leaves the other anatomical structures of the posterolateral corner (i.e., popliteal ligament, posterolateral ligament, arcuatum ligament, and gastrocnemius lateral head) intact provides joint stability and satisfactory functional outcomes. To our knowledge, this is the first study to assess knee flexion strength following proximal fibula resection, with results indicating good post-resection strength compared to the contralateral knee. Some studies suggest that the knee retains functional stability following proximal fibula resection as the remaining stabilizing structures of the knee remain intact, making LCL reconstruction unnecessary [14]. Multiple techniques, such as the re-attachment to the lateral metaphysis of the tibia or the re-attachment to nearby capsular and ligamentous tissues, as well as no reconstruction at all, have been reported in the literature [15]. Zhao et al. compared different reconstruction methods of the posterolateral corner following proximal fibula resection: (1) anchoring the LCL and BFT to the lateral tibial metaphysis versus (2) no reconstruction. They found superior outcomes in terms of stability and clinical scores in the reconstruction group, with 0 cases of instability out of 12, compared to 3 cases with signs of instability out of 7 in the non-reconstruction group as well as a median MSTS of 93% and 87%, respectively [16]. Similarly to our study, Arikan et al. investigated knee instability after proximal fibula resection in six Malawer type I resections using a different surgical technique by re-attaching the LCL and the BFT to the surrounding soft tissue [14]. Four out of six patients showed grade 1 joint opening, while two out of six displayed grade 2 joint space opening. In our study, none of the six cases demonstrated grade 2 joint space opening, indicating that the attachment to the posterolateral knee soft tissue might not be sufficient due to the high forces involved in knee flexion and joint stability. Our results suggest that a fixation via the bone anchoring of the LCL and BFT provides good results regarding stability and strength, with the decartilaged former PTFJ serving as the primary landmark for anchor insertion. Arikan et al. also observed one persistent peroneal paresis as well as high MSTS Scores ranging from 86 to 100%, which is similar to our findings and in line with previous published literature [1,14,15,16,17,18,19,20]. Iatrogenic peroneus paresis in proximal fibula resection has been described in up to 57% of patients [17,20]. The patient in the current study who developed persistent peroneus paresis was operated on multiple times, receiving two previous intralesional curettages before being admitted to our department, undergoing a proximal fibula resection. The loss of peroneal function results in significantly lower functional outcomes, with the affected patients showing the lowest MSTS value of 70%. With ACT and GCT, two different entities of tumors were observed in our case series. As these are two of the most common types of tumors to occur in the proximal fibula, this does not contradict the existing literature [2]. Recurrence rates are described, with 10–40% for GCT and 0–26% for ACT [21,22]. In 2010, Abdel et al. conducted a study comparing recurrence rates based on surgical techniques for proximal fibula tumors [23]. Their research involved 121 benign tumors, treated with intralesional curettage, marginal excision, or a type I resection. The overall recurrence rate was 8% (10 cases), with a significantly higher recurrence for the intralesional curettage group compared to the type 1 resection group (23% vs. 5%, *p* = 0.029). When analyzed by tumor entity, GCTs and aneurysmal bone cysts accounted for the majority of recurrences. With minimizing the risk for recurrence in mind, our observations favor a proximal fibula resection over an intralesional curettage in controversial cases as the re-attachment of the posterolateral structures provide satisfactory clinical outcomes, no significant loss of knee flexion strength, and seem to not cause major morbidity.

## 5. Conclusions

Malawer type I en bloc resections are a valid treatment option for aggressive benign tumors, with low recurrence rates. The posterolateral corner necessitates reconstruction in proximal fibula resection due to its complex biomechanical role in knee stability. Utilizing bone anchors at the decartilaged former PTFJ results in promising clinical and functional outcomes, as well as favorable subjective patient reports. Anatomical challenges and possible postoperative complications must be considered.

## Figures and Tables

**Table 1:** Case report form of the included patients. Legend: F—female, M—male, ACT—atypical cartilaginous tumor, and GCT—giant cell tumor of bone.

Case N°	Year of Birth	Age at OP (Years)	Gender	Diagnosis	Follow Up (Months)	MSTS Score (Points, /30)	Knee Flexion Power (Operated/Non Operated Limb, Percent)	Lateral Stability (Grade, Modified Hughston Classification)
1	1972	48	F	ACT	23	29	5.3/6.3, 84.1%	1
2	1997	22	F	GCT	29	30	9.1/8.8, 103.4%	1
3	1993	27	M	GCT	14	21	10.1/12.2, 81.9%	1
4	1983	37	M	ACT	16	30	8.1/9.9, 81.8%	1
5	1978	42	F	ACT	19	29	11.8/12.3, 95.9%	1
6	1961	59	F	ACT	14	28	2.8/2.9, 96.5%	1

## List of Abbreviations

GCT	Giant cell tumors
ACT	Atypical cartilaginous tumors
PTFJ	Proximal tibiofibular joint
LCL	Lateral collateral ligament
BFT	Biceps femoris tendon
ROM	Range of motion
MSTS	Musculoskeletal Tumor Society Score
